# Middle Meningeal Artery Embolization for the Treatment of Bilateral Chronic Subdural Hematoma

**DOI:** 10.3389/fneur.2021.651362

**Published:** 2021-10-28

**Authors:** Qi Wei, Gangxian Fan, Zhenzhu Li, Qingbo Wang, Ke Li, Chao Wang, Zefu Li

**Affiliations:** ^1^Department of Neurosurgery, Binzhou Medical University Hospital, Binzhou, China; ^2^Department of Neurosurgery, Linyi People's Hospital, Linyi, China; ^3^Department of Neursosurgery, Hwa Mei Hospital, University of Chinese Academy of Sciences, Ningbo, China; ^4^Ningbo Institute of Life and Health Industry, University of Chinese Academy of Sciences, Ningbo, China

**Keywords:** hematoma, subdural, chronic, meningeal arteries, interventional embolization, neurosurgery

## Abstract

**Background:** Bilateral chronic subdural hematoma (bCSDH) is a frequent condition commonly linked to the need for retreatment; however, the reason for this high retreatment rate remains unclear. The middle meningeal artery (MMA) was found to have a relationship with the occurrence and development of chronic subdural hematomas. This study examines a possible method to reduce bCSDH recurrence using bilateral MMA embolization combined with bilateral burr-hole drainage.

**Materials and Methods:** Ten patients with bCSDH who underwent bilateral MMA embolization combined with bilateral burr-hole drainage at our hospital between June 2018 and May 2020, were retrospectively analyzed. Patients' clinical information, prognoses, imaging results, as well as surgical results were documented and analyzed.

**Results:** Ten patients were diagnosed with bCSDH with no comorbid brain diseases. They underwent bilateral MMA embolization combined with bilateral burr-hole drainage. We embolized the MMA immediately before burr hole drainage successfully and employed angiography to validate these results. All the patients attained relief of symptoms without adverse events, and no re-expansion or relapse was reported in the follow-up computed tomography.

**Conclusion:** Bilateral MMA embolization combined with bilateral burr-hole drainage is an available treatment for patients with bCSDH and may have the potential for preventing recurrence.

## Introduction

Chronic subdural hematoma (CSDH) is among the most frequent conditions, with incidence rates varying from 5.3 to 13.5 cases per 100,000 person years in the general population and with a higher incidence in the older population (1). Generally, CSDH is regarded a delayed sequela of head trauma, resulting in the infiltration of phagocytic macrophages and neovascular proliferation at the site of hematomas (2). CSDH is encountered frequently in neurology clinics. Unilateral CSDH (uCSDH) occurs in a remarkable number of individuals with CSDH, however bilateral CSDH (bCSDH) is occasionally encountered in neurosurgical practice. Cases of bCSDH, which account for about 14–25% of all CSDH, are believed to be a distinct subgroup from uCSDH (3–5). Presently, surgical evacuation with craniotomy or burr holes constitutes the main treatment of symptomatic CSDH (6, 7). In researches on CSDH treatment with burr-hole surgery, 11–33% of operative cases experienced a relapsed hematoma (8–10). Initially, most clinicians considered bCSDH as equivalent to uCSDH since there was no distinct difference in either disease presentation or treatment strategy. Non-etheless, research evidence has documented that bCSDH cases were linked to increased retreatment rates (4, 11). Others have also documented that bCSDH was an independent predictor of CSDH relapse although the cause of this elevated rate of relapse remains unclear (11, 12).

The middle meningeal artery (MMA) may play an indispensable role in CSDH onset and progress. As reported in the literature, there was a rich blood vessel network on the dura mater, and the blood supply of the dura mater at the fronto-tempo-parietal area was mainly derived from the branch of the external carotid artery-the MMA (13–16). Histological investigations have illustrated that some small blood vessels from the MMA were found to be connected to the CSDH outer membrane through the dura mater (6, 17–20). Therefore, embolization of MMA might be a beneficial strategy for treating refractory or recurrent CSDH. Recently, MMA embolization has been described as an alternative treatment for relapsed or primary CSDH. Studies on performing MMA embolization for treating individuals with CSDH have also been published (17, 21, 22). Embolization of the MMA was found to be efficient for individuals with refractory CSDH or CSDH with relapse risk, and was reported as a beneficial treatment approach to avert enlargement of hematoma (23).

Despite some researches reporting the use of MMA embolization for treating individuals with CSDH, none, to our knowledge, have documented on the effect of bilateral MMA embolization as a pre-treatment strategy for bCSDH to prevent the associated high recurrence rate. Herein, we document on the effective utility of bilateral MMA embolization combined with bilateral burr-hole drainage to treat patients with bCSDH.

## Materials and Methods

### Patient Selection

Data for 10 individuals with bCSDH treated at our center from June 2018 to May 2020 were analyzed retrospectively. The inclusion criteria were: (A) all patients clearly diagnosed with bCSDH via brain computed tomography (CT); (B) patients with symptomatic bCSDH; and (C) >14 days must have elapsed from the time of head trauma to the time of performed operation. The exclusion criteria consisted of: (a) CSDH cases with comorbid conditions (vascular lesions, brain tumor, arachnoid cyst, spontaneous intracranial hypotension, or previous craniotomy); (b) poor medical condition with life expectancy of <6 months. The study approval was granted by the ethics committee of Binzhou Medical University Hospital (No. 2018065). All individuals analyzed in this study provided informed consent. The study protocol adhered to the Declaration of Helsinki.

### Clinical Evaluation

The clinical data of 10 patients were documented, including their basic information such as age and gender, clinical manifestation, operation method and time, and follow-up time. The GCS (Glasgow Coma Scale) was employed to determine the preoperative neurological function. The collected data were checked, unified, and summarized into a table form.

### MMA Embolization

A local anesthetic was administered prior to bilateral MMA embolization. Afterwards, Seldinger's technique was employed to puncture the femoral artery. A standard 6-French sheath was utilized to gain entry into the femoral artery. The guiding catheter was placed in the external carotid artery. Next, angiography of the external carotid artery was conducted, in which an Excelsior^®^ SL-10 microcatheter (Target Therapeutics/Boston Scientific, Fremont, California, USA) was placed under the guidance of fluoroscopy and the Traxcess^®^ 14 microguidewire (MicroVention, California, USA) was placed into the trunk both middle meningeal arteries respectively (10, 23). Under blank fluoroscopic roadmap control, embolization was performed by coiling to the embolization site through the microcatheter. Several types of coils were used: Detachable coils (Target^®^ detachable coil, Boston Scientific, California, USA), Microplex (Microvention, California, USA), and Axium (ev3, California, USA). Next, we confirmed these results by intraoperative angiography, the guiding catheter along with the femoral arterial sheath were removed, followed by placing of the occlusion in the puncture site of the femoral artery. The all patients was treated using MMA embolization before burr-hole drainage to mitigate the symptoms, promote prognosis, as well as decrease the chances of relapse. After the operation, we moved all the study participants into the ward for observation.

### Follow-Up

On the admission of each participant, a cranial CT scan was employed to diagnose hematomas, with the imaging conducted once every week or as per the changes in clinical symptoms as needed by each individual patient during hospitalization. Incidences of post-surgery acute intra-cranial bleeding after treatment of bCSDH using surgery were documented. Upon improvement of the symptoms, all the study subjects were discharged. A brain CT once in a month was recommended and a 3–4 month follow-up was performed for all the participants. The GOS (Glasgow Outcome Scale) along with mRS (modified Rankin Scale) were employed to evaluate the post-surgery neurological function at the final clinic visit. When the symptoms worsened or reappeared, the involved patient was re-admitted. Recurrence of CSDH was defined as patients who had worsening symptoms as illustrated by the CT-verified re-accumulation of CSDH at- or near an initial hematoma site post the initial surgery, as well as those who needed a repeat surgery (3).

## Results

A summary of the baseline features of the study participants is provided in Table 1. All the 10 study subjects were men (mean age = 63.1 years; range 40–76 years). All the 10 study subjects had a head trauma history, and the GCS was higher than 13 on the admission date for all participants. The most common symptoms among the subjects included headache, nausea, vomiting, limb weakness, unsteady gait, and slurred speech. The imaging data of all the participants were verified prior to the operation. A summary of the surgical methods and time of the study subjects, and the follow-up time is provided in Table 2. All the study subjects were treated with bilateral MMA embolization combined with bilateral burr-hole drainage. Embolization was successful in all the participants, with no adverse event reported, and bleeding was promptly stopped. No rebleeding occurred during the 3–4 month follow-up as evidenced by the brain CT scans. Moreover, the size of the hematoma cavity decreased gradually, thereafter.

**Table 1:** Baseline characteristics and prognoses of patients with bilateral chronic subdural hematoma.

**Characteristics**		**No. of patients *N* = 10**
Age (year)	40≤, <60	2
	≥60	8
Hematoma location	^a^b-Parietal	2
	b-Fronto-parietal	2
	b-Fronto-parieto-temporal	6
Clinical symptoms	Headache	6
	Nausea, vomiting	3
	Slurred speech	1
	Unsteady gait	2
	Weakness in both lower limbs	2
^b^Drug therapy	Yes	1
	No	9
^c^GCS	14	2
	15	8
^d^GOS	0–IV	0
	V	10
^e^mRS	0	6
	1	4

a *b, bilateral;*

b *Drug therapy, current anticoagulant or antiplatelet therapy;*

c *GCS, Glasgow Coma Scale;*

d *GOS, Glasgow Outcome Scale;*

e *mRS, modified Rankin Scale*.

**Table 2:** The patient's operation method, operation time and follow-up time.

**Patient**	**Operation method**	**Time of MMA^a^ embolization (min)**	**Time of burr hole dranage (min)**	**Follow-up time (days)**
1	MMA embo and burr holes^b^	55	60	106
2	MMA embo and burr holes	48	63	97
3	MMA embo and burr holes	60	72	122
4	MMA embo and burr holes	52	65	115
5	MMA embo and burr holes	50	70	120
6	MMA embo and burr holes	45	68	98
7	MMA embo and burr holes	58	75	115
8	MMA embo and burr holes	54	64	130
9	MMA embo and burr holes	55	68	108
10	MMA embo and burr holes	52	66	112

a *MMA, middle meningeal artery;*

b *MMA embo and burr holes: bilateral MMA embolization in combination with bilateral burr hole drainage*.

### Representative Cases' Clinical Data

#### Patient 1

**Figure 1:**
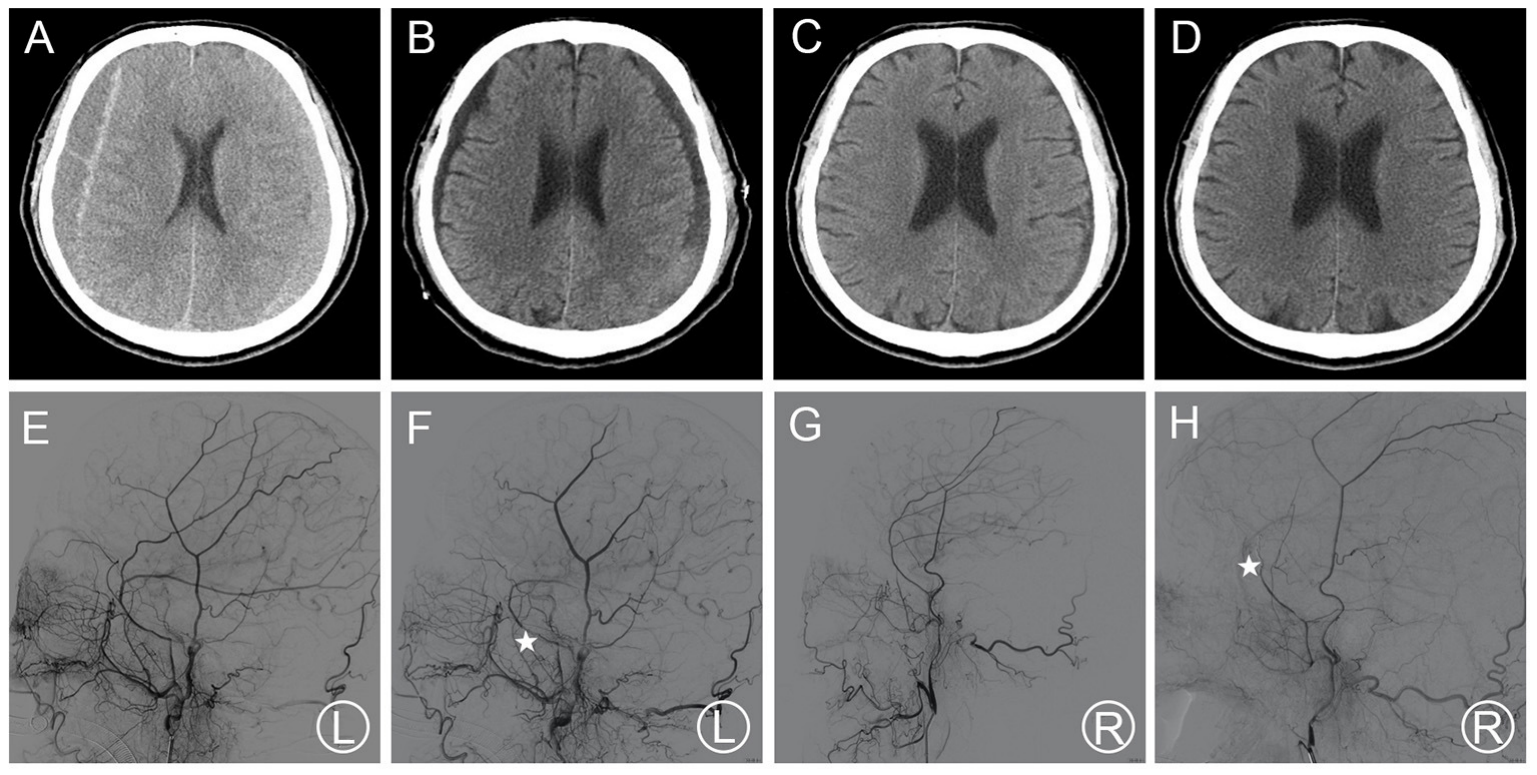
**(A)** Brain CT images of a hematoma in patient 1 before treatment. **(B)** Brain CT was repeated before the patient's discharge. **(C,D)** Follow-up brain CT was performed at 35 and 115 days. **(E,F)** Pre- and post-operative DSA presented left MMA and the left MMA disappeared (asterisk). **(G,H)** Pre- and post-operative DSA presented right MMA and the right MMA disappeared (asterisk). DSA, digital subtraction angiography; CT, computed tomography; MMA, middle meningeal artery.

Patient 1 was treated for coronary heart disease with oral aspirin. He had minor head trauma history 25 days before and visited a neighborhood hospital with a complaint of headache. He underwent conservative treatment and regular follow-ups were requested. The patient was not required to stop aspirin medication owing to the high risks associated with its discontinuation. Head CT performed 25 days after trauma showed a slight enlargement of the bilateral hematoma and increased density (Figure 1A). Meanwhile, his symptoms became more serious as the headache worsened, and he developed a new symptom of vomiting. These results were regarded as aggravation signs. Thus, bilateral MMA embolization combined with bilateral burr-hole drainage was advised and performed under local anesthesia in our hospital. Cerebral angiography was conducted pre-, as well as post-surgery for MMA embolization (Figures 1E–H). As proof of proper embolization, the MMA could not be observed during post-surgery angiography. His symptoms improved postoperatively. A repeat brain CT scan exhibited no re-bleeding prior to discharge, and the hematoma cavity gradually decreased in size (Figures 1B–D). No recurrence was observed from discharge to 3–4 months after discharge.

#### Patient 2

**Figure 2:**
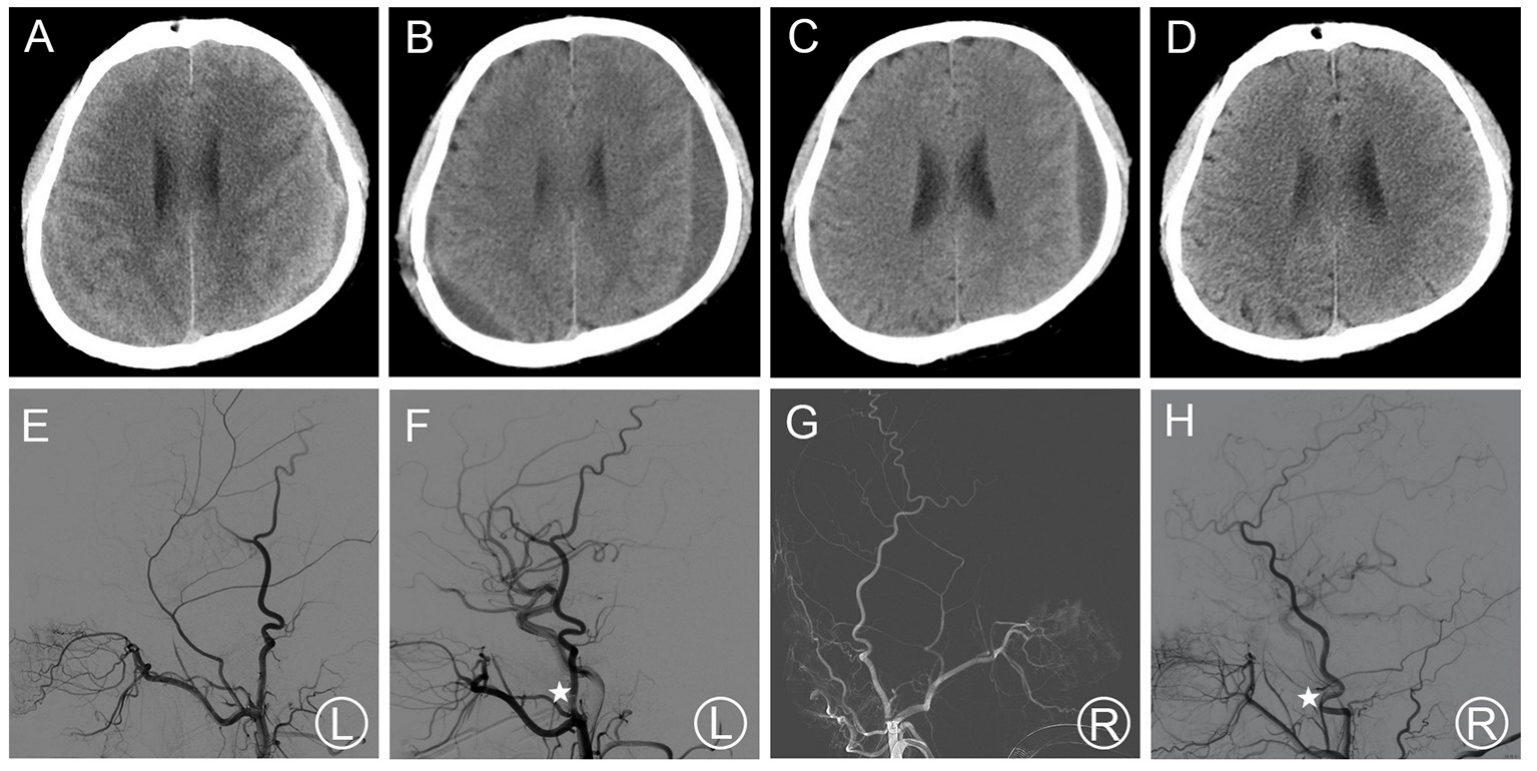
**(A)** Brain CT images of a hematoma in patient 2 before treatment. **(B,C)** Brain CT was repeated at 14 days after surgery and before the patient's discharge. **(D)** Follow-up brain CT was performed at 97 days. **(E,F)** Pre- and post-operative DSA presented left MMA and the left MMA disappeared (asterisk). **(G,H)** Pre- and post-operative DSA presented right MMA and the right MMA disappeared (asterisk). DSA, digital subtraction angiography; CT, computed tomography; MMA, middle meningeal artery.

Patient 2 was admitted to a neighborhood hospital after experiencing a fall, hence received a hard blow to the back of the head. Within 20 days, his follow-up head CT scan revealed thin bCSDH. The patient received conservative treatment and was followed up. Since bilateral hematoma enlargement was observed 20 days later (Figure 2A), and the patient had slurred speech, he visited our hospital. MMA embolization in combination with bilateral burr-hole drainage was suggested under local anesthesia. Cerebral angiography was carried out pre-, as well as post-surgery for MMA embolization (Figures 2E–H). The MMA could not be observed during postoperative angiography. The symptoms of the patient improved post-surgery. Likewise, a repeat CT scan of the brain revealed no re-bleeding prior to discharge. In addition, the hematoma cavity size decreased gradually, thereafter (Figures 2B–D). No recurrence was observed from discharge to 3–4 months after discharge.

## Discussion

CSDH is among the most frequent diseases. Its initiation and progress involves complex processes. During this process, the histology of the outer membrane has been the focus of investigation on the mechanisms by which CSDH develops (24). Investigations have showed that the neovascular blood supply of the CSDH outer membrane was mainly MMA (25, 26). These findings suggested the possibility of treating CSDH through MMA embolization. Herein, we prevented bCSDH recurrence by embolizing the MMA bilaterally, and no hematoma recurrence was found during the follow-up period after embolization.

Currently, bCSDH is thought to be a distinct subgroup from uCSDH (27). The clinical presentation of bCSDH shows greater diversity than that of uCSDH. Tsai et al. (28), comparing the clinical manifestations in unilateral vs. bilateral CSDH, documented that individuals with bCSDH had a high chance of exhibiting symptoms linked to elevated intracranial pressure, consisting of headache, nausea, vomiting and brain herniation, while individuals with uCSDH highly likely exhibited symptoms linked to brain shift, consisting of hemiparesis, which might be linked to the loss of counterbalance related with a unilateral hematoma. Additionally, bCSDH sometimes may be asymptomatic, resulting in a delayed or missed diagnosis because of less specific clinical presentation. Furthermore, bCSDH generally has a poorer clinical outcome and progresses more rapidly than uCSDH. Although a previous research found no significant differences in postoperative outcomes between the bilateral and unilateral CSDH groups (4), in more recent studies, outcomes were poorer in the bCSDH group (3, 5, 29, 30), with bilateral hematoma cited as having an elevated risk of acute aggravation of clinical manifestation due to downward herniation. Therefore, early and rapid surgical drainages of bilateral hematomas are needed to achieve better outcomes and recovery of neurological function and may be performed with success rates similar to those in unilateral hematomas (5, 31, 32).

Patients with CSDH generally have a good prognosis; however, the postoperative recurrence rate ranges from 11 to 33% (8). Investigations on recurrence factors have always been a hot spot in CSDH research. Several predictors of CSDH recurrence have been documented consisting of advanced age, arachnoid cyst, alcohol abuse, persistent midline shift, the utilization of perioperative antiplatelet/anticoagulant treatment, bleeding tendency, presence of thick membranes, bilateral CSDH, brain atrophy, lower GCS scores, hematoma density, inflammation markers, poor post-surgery brain re-expansion, post-surgery subdural air aggregation, as well as some technical surgery aspects (9, 12, 28, 33–36). However, some results have not yet been confirmed, and some of these predictors appear to play different roles in bCSDH and uCSDH development. Studies have indicated that the mean age of bCSDH patients was significantly greater than that of uCSDH patients. Old age is a known predictor of CSDH development and an age of more than 75 years has been documented to be an independent predictor of bCSDH development (3, 37). This might be linked to brain atrophy along with increased venous fragility. Although persistent brain shifting was described as the current mainstream theory of recurrence, leading to tearing of the bridging veins because of a lack of surrounding reinforcement, the pathogenesis of recurrent CSDH cannot be explained satisfactorily by a single theory (1, 11, 31). These findings may help identify patients at a high risk of CSDH recurrence and assist in developing personalized treatment options to reduce recurrence.

Presently, there is no uniform standard for treating bCSDH, and it has a higher recurrence rate than uCSDH regardless of whether it was managed via unilateral or bilateral surgery. One study found unilateral surgery for bCSDH to be an independent risk factor for retreatment (1). This study reported that the retreatment rate for individuals under bilateral surgery treatment was 14.1% (18 of 128 cases), which was remarkably lower relative to the retreatment rate in individuals under unilateral surgery treatment (28.7%; *P* < 0.004). Although unilateral surgery is still very commonly performed for bCSDH, especially when the contralateral hematoma is asymptomatic and thin, after a certain period, the contralateral hematoma might enlarge, causing symptoms, requiring another burr-hole surgery (30, 38). Our previous study reported that bCSDH worsened on the contralateral side after unilateral surgery in two patients (39). This phenomenon may be explained by the decrease in the intracranial pressure owing to the unilateral evacuation, permitting expansion of the non-operated contralateral hematoma. Unilateral drainage could also result in the displacement of the brain toward the operated side, expanding the contralateral subdural space (1, 5, 30). Furthermore, Lin et al. (31) found that the incidence of the late phase of brain shifting post-evacuation was remarkably greater in bCSDH in contrast with uCSDH, which they proposed might be one factor responsible for the higher symptomatic recurrence rate in patients with bCSDH.

To reduce the recurrence rate of bilateral hematomas, bCSDH should be differentiated from unilateral cases to establish an appropriate surgical strategy. Presently, some studies have reported on small vessel connections between the MMA and outer membranes of CSDH, leading to a new approach to interventional therapy for CSDH, which is MMA embolization (17, 20, 40). Link et al. (6) introduced a novel approach to MMA embolization, which provides a minimally invasive and low-risk initial treatment alternative to surgery for patients with symptomatic CSDH when clinically appropriate. In our previous study (41), we reported on the use of absolute alcohol as an embolic agent to prevent the re-expansion and relapse of acute epidural hematoma and CSDH *via* MMA embolization; the hematomas of all patients gradually decreased and disappeared after the procedure, with no instances of re-expansion nor recurrence of the hematoma during follow-up. Although studies have shown that the use of any embolic material (liquid or solid) could achieve the desired effect for the occlusion of the MMA, liquid embolic agents needed to pay attention to whether there is anastomotic branches between the internal and external carotid arteries to prevent the liquid embolic agent flowed into the brain and caused inappropriate embolism (6, 21). Therefore, the solid embolic material was safer without excluding this dangerous anastomosis. In our study, 10 patients with bCSDH were treated using bilateral prophylactic embolization of the MMA combined with bilateral burr-hole drainage. Embolization was performed to reduce intraoperative bleeding and postoperative hematoma recurrence. Surgical drainage was performed to alleviate symptoms. For all study subjects, symptoms resolved postoperatively, and they were discharged after showing symptomatic improvement. Follow-up brain CT was performed, and no patients experienced hematoma recurrence for at least 3 months after discharge.

There are several limitations to our study. First, this was a retrospective study with a relatively small sample size and without a control group. Second, data were collected through medical records and imaging reviews and were thus less accurate compared to a hypothetical prospective study. Third, the evaluation period for neurological outcomes varied and follow-up information was difficult to obtain without scheduled returns to the clinic. Despite these miscellaneous limitations, our study provides useful preliminary information to identify both the clinical features of bCSDH and to assess a new treatment idea to reduce its high recurrence rate.

## Conclusions

In summary, although there were similarities between bCSDH and uCSDH case features, it is known that bCSDH is more likely to recur than uCSDH. Thus, bCSDH treatment requires clinical differentiation from uCSDH to establish an appropriate surgical strategy. Herein, bilateral MMA embolization combined with bilateral burr-hole drainage was an available treatment for patients with bCSDH with the potential for preventing recurrence.

## Data Availability Statement

The original contributions presented in the study are included in the article/supplementary material, further inquiries can be directed to the corresponding authors.

## Ethics Statement

The study was reviewed and approved by the Ethics Committee of Binzhou Medical University Hospital (No. 2018065). The patients/participants provided their written informed consent to participate in this study.

## Author Contributions

QW and GF designed and carried out the research, as well as wrote the manuscript. ZeL designed the research, performed report supervision, and revised the article critically for content. CW designed the research and contributed to the analysis. ZhL, QW, and KL participated in the acquisition, analysis, and interpretation of the data and provided clinical advice. All authors contributed to the article and approved the submitted version.

## Funding

This research was funded by Binzhou Medical University Scientific Research Foundation (BY2018KJ02 and BY2016KYQD15) along with Shandong Province Natural Science Foundation of China (ZR2018LH007 and ZR2017LH033).

## Conflict of Interest

The authors declare that the research was conducted in the absence of any commercial or financial relationships that could be construed as a potential conflict of interest.

## Publisher's Note

All claims expressed in this article are solely those of the authors and do not necessarily represent those of their affiliated organizations, or those of the publisher, the editors and the reviewers. Any product that may be evaluated in this article, or claim that may be made by its manufacturer, is not guaranteed or endorsed by the publisher.
